# Supplementary material for: Mapping Asia Plants: The Threat Status and Influencing Factors of Rare and Endangered Vascular Plant Species in North Asia (Asian Russia)
Source: Plants (Basel). 2023 Jul 27;12(15):2792. doi: 10.3390/plants12152792 (PMC10421321; doi:10.3390/plants12152792)
Supplement: Supplementary file 1 [file plants-12-02792-s001.zip › Supplementary Materials File S2.pdf]

Supplementary Materials File S2: A comparison of IUCN criteria with ones in the Red Data Book of the Russian Federation [2008]

**Status category scale for assessing the state of species listed in IUCN (https://www.iucnredlist.org/)**	**Comments at comparison**	**Status category scale for assessing the state of plant and fungal species listed in the Red Data Book of the Russian Federation (2008)**
E (Extinct)	E (IUCN) corresponds to category "0" of the Red Data Book of the RF (2008). It may also correspond to the category "0" of a regional Red Book, if the taxon also disappeared on the territory of the country (usually refers to taxa found only in 1-3 regions).	0 - The species (subspecies) may be extinct in the region, which has not been in nature for a long time (at least – 50 years), but the possibility of their retention cannot be ruled out.
EW (Extinct in the Wild)	EW – this criterion is absent as a special category in the documentation for the Red Data Book of the RF (2008).	-
RE (Regionally Extinct)	RE (IUCN), as a rule, corresponds to the category "0" of a regional Red Book, provided that the taxon, having disappeared in this region, has been preserved in other regions of the country.	-
CR (Critically Endangered)	Category CR corresponds to the category "1" very closely.	1- Endangered. Taxa whose numbers have been reduced to such a level or the number of their localities has been so reduced that they may soon become extinct.
EN (Endangered)	Category EN has no Russian counterpart. In fact, it partly consists of taxa of category “1”, partly of category “2”.	-

	Although we saw interval categories in some Red Books: for example, “1-2” or “2-3”, such categories are not provided for by federal documents even at the regional level.	
VU (Vulnerable)	Category VU, as a rule, corresponds to the Russian category "2"	2- Decreasing in numbers. Taxa with steadily declining numbers, which, with further exposure to factors that reduce the number, can quickly fall into the category of endangered.
-	-	2 a - taxa whose numbers are declining as a result of changing living conditions or destruction of habitats;
-	-	2 b - taxa that are declining as a result of human overuse and may be stabilized by special protection measures (medicinal, food, ornamental and other plants).
NT (Near Threatened)	The NT category generally corresponds to "3" category of the Red Data Book of the RF (2008). This category, according to the IUCN criteria, usually includes taxa that, according to one or more quantitative criteria, are close to the boundaries established for classifying a taxon in the VU category, but do not yet allow the taxon to be classified in this category.	3- Rare. Taxa with a natural low abundance, found in a limited area (or water area) or sporadically distributed over large areas, for the survival of which the adoption of special conservation measures is necessary:
-	-	3 a - narrow endemics;
-	-	3 b - having a significant range, within which they occur sporadically and with a small number of populations;

-	-	3 c - having a narrow ecological confinement associated with specific growing conditions (outcrops of limestone or other rocks, saline soils, littoral habitats, etc.);
-	-	3 d - having a significant common area, but located within Russia at the border of distribution;
-	-	3 e - having a limited range, part of which is located on the territory (or water area) of Russia.
DD (Data Deficient)	Under the IUCN procedure, this assessment is given before the taxon is classified as protected, and in the Russian Red Data Book, as a rule, after.)	4 - Uncertain by status. Taxa that probably belong to one of the previous categories, but there is currently no sufficient information about their state in nature, or they do not fully meet the criteria of other categories, but need special protection measures.
-	Russian category "5" is not in the IUCN documents, but it can be classified as an LC category.	5 - Restorable and recoverable. Taxa, the number and area of distribution of which have begun to recover, under the influence of natural causes or as a result of conservation measures, and they are approaching a state where no special conservation and restoration measures are needed.
LC (Least Concern)	In Russian practice (Red Data…, 2008) these are taxa that do not deserve to be included in the Red Book, and, consequently, special protection	-
NE (Not Evaluated)	-	-
